# Cryo-electron tomography of periplasmic flagella in *Borrelia burgdorferi* reveals a distinct cytoplasmic ATPase complex

**DOI:** 10.1371/journal.pbio.3000050

**Published:** 2018-11-09

**Authors:** Zhuan Qin, Jiagang Tu, Tao Lin, Steven J. Norris, Chunhao Li, Md A. Motaleb, Jun Liu

**Affiliations:** 1 Department of Microbial Pathogenesis, Microbial Sciences Institute, Yale University, New Haven, Connecticut, United States of America; 2 Department of Pathology and Laboratory Medicine, McGovern Medical School, Houston, Texas, United States of America; 3 Philips Research Institute, School of Dental Medicine, Virginia Commonwealth University, Richmond, Virginia, United States of America; 4 Department of Microbiology and Immunology, Brody School of Medicine, East Carolina University, Greenville, North Carolina, United States of America; University of Texas Southwestern Medical Center, UNITED STATES

## Abstract

Periplasmic flagella are essential for the distinct morphology and motility of spirochetes. A flagella-specific type III secretion system (fT3SS) composed of a membrane-bound export apparatus and a cytosolic ATPase complex is responsible for the assembly of the periplasmic flagella. Here, we deployed cryo-electron tomography (cryo-ET) to visualize the fT3SS machine in the Lyme disease spirochete *Borrelia burgdorferi*. We show, for the first time, that the cytosolic ATPase complex is attached to the flagellar C-ring through multiple spokes to form the “spoke and hub” structure in *B*. *burgdorferi*. This structure not only strengthens structural rigidity of the round-shaped C-ring but also appears to rotate with the C-ring. Our studies provide structural insights into the unique mechanisms underlying assembly and rotation of the periplasmic flagella and may provide the basis for the development of novel therapeutic strategies against several pathogenic spirochetes.

## Abstract 2

Cryo-electron tomography of periplasmic flagella in the Lyme disease bacterium Borrelia burgdorferi reveals it to have a distinct cytoplasmic ATPase complex and an atypical interaction with the flagellar C-ring.

## Author summary

Type III secretion systems are widely utilized by gram-negative bacteria to assemble flagella or to transport virulence effectors into eukaryotic cells. The central component is known as a type III secretion machine, which consists of a membrane-bound export apparatus and a cytosolic ATPase complex. Powered by the proton motive force and ATP hydrolysis, the secretion machine is responsible for substrate recognition and export. Here, we use the Lyme disease spirochete *B*. *burgdorferi* as a model system to unveil unprecedented structural details of the intact flagellar secretion machine by high-throughput cryo-electron tomography (cryo-ET) and subtomogram averaging. We provide the first structural evidence that the cytosolic ATPase complex is attached to the flagellar C-ring through multiple spokes to form the “spoke and hub” structure in *B*. *burgdorferi*. The novel architecture of the ATPase complex not only strengthens the flagellar C-ring but also enables an optimal translocation of substrates through the ATPase complex and the export apparatus.

## Introduction

A group of bacteria named spirochetes can cause serious human diseases such as Lyme disease (*Borrelia* or *Borreliella* species), syphilis (*Treponema pallidum* subsp. *pallidum*), and leptospirosis (*Leptospira interrogans* and other *Leptospira* species). Spirochetes are easily recognized by their distinctive wave-like or helical morphology and unique modes of motility. Recent genetic studies indicate that their motility is crucial for host infection and/or bacterial transmission [1–6]. Spirochetal motility is driven by periplasmic flagella, which reside and rotate between the outer membrane and the peptidoglycan layer. Mutant *B*. *burgdorferi* cells that lack their periplasmic flagellar filaments are nonmotile and rod shaped [1,2,7–9].

Similar to the flagella in the model organisms *Escherichia coli* and *Salmonella enterica*, periplasmic flagella are composed of the flagellar motor, the hook, and the filament. However, the periplasmic flagella are noticeably different from other bacterial flagella in several aspects. The spirochetal flagellar motor is significantly larger than those in *E*. *coli* and *S*. *enterica* (approximately 80 nm versus approximately 45 nm in diameter). A periplasmic “collar” contributes significantly to the motor structures observed in *B*. *burgdorferi* [10,11] and all other spirochetes characterized to date [12–15]. The large flagellar motor from *B*. *burgdorferi* appears to produce the highest torque (approximately 4,000 pN nm) observed in bacteria [16]. Furthermore, spirochetes have unusual flagellar hooks in which the hook proteins are cross-linked by a covalent bond, which is required to transmit the torque from the motor to the filament [17]. Those spirochete-specific features enable the spirochetes to bore through viscous environments in their animal hosts.

The filament is the largest component of the periplasmic flagella. Multiple filaments arising from both poles form flat ribbons that wrap around the spirochete cell body in a right-handed fashion [7]. The flagella filament is assembled by the flagellar-specific type III secretion system (fT3SS), which is conserved across different bacterial species [14,18]. Additionally, the fT3SS is evolutionally related to the virulence T3SSs (vT3SSs) that promote bacterial virulence by delivering effector proteins into eukaryotic cells [19,20]. The fT3SS is powered by proton motive force [21–23] or sodium motive force [24], with additional involvement of ATP hydrolysis [25–27].

The fT3SS consists of a membrane-bound export gate complex made up of six membrane proteins (FlhA, FlhB, FliO, FliP, FliQ, and FliR) and a large cytosolic ATPase complex formed by three cytoplasmic proteins (FliH, FliI, and FliJ). The ATP complex promotes the export process by binding and delivering substrates to the export apparatus [28,29]. FliI is an ATPase and shows structural similarity with the α and β subunits of the F_O_F_1_–ATP synthase [30]; it exhibits its full ATPase activity when it self-assembles into a homohexamer [27,31]. FliH probably acts as a negative regulator of the FliI ATPase, and FliJ has chaperone-like activities [28,32]. FliH, FliI, and FliJ coordinately deliver a chaperone–substrate complex to the export gate by binding to the docking platform of the fT3SS for substrate export [33]. FliH_2_ binds to the FliI ATPase and localizes FliI to the bottom of the flagellar motor through the interaction with FliN on the C-ring [34,35]. FlhA is required for stable anchoring of the FliI_6_ ring to the gate [36]. FliP, FliQ, and FliR form an export gate complex with helical symmetry [37]. Cryo-electron tomography (cryo-ET) studies have revealed the overall structures of the fT3SS machines in intact flagella [11–15,38–40]. However, those studies have not yet provided sufficient details on stoichiometry or architecture to fully understand the components of the ATPase complex and its interactions with other proteins of the flagellar motor.

*B*. *burgdorferi* is the best-studied spirochete model system. Recent breakthroughs in genetic manipulations allow the production of well-defined mutations without imposing any secondary alterations [2,10,39,41]. The small cell diameter and the highly ordered array of multiple flagellar motors at cell poles make *B*. *burgdorferi* an excellent system for in situ structural analysis of the periplasmic flagella and their fT3SS machines by cryo-ET. Our previous structural analysis of wild-type (WT) cells and several rod mutants of *B*. *burgdorferi* revealed the sequential assembly of the flagellar rod, hook, and filament [39]. Furthermore, disruption of the *fliH* and *fliI* genes by transposon mutagenesis was found to disrupt the assembly and placement of the cytoplasmic ATPase complex and to greatly inhibit flagellar filament formation, which were largely restored by genetic complementation [42].

In this study, we used cryo-ET and subtomogram averaging to reveal novel features of the ATPase complex in the WT *B*. *burgdorferi* periplasmic flagellar motor. The ATPase complex is attached to the C-ring by spokes, and without the spokes, the C-ring became more flexible and elliptical in shape. Furthermore, we resolved the symmetry mismatching between the stators and spokes in class averages, showing that the ATPase complex and C-ring rotate as a rigid body with respect to the stators and collar. Comparing these results with recent studies of the T3SSs in external flagella and evolutionarily related injectisomes provides new insights into these nanomachines that are structurally and functionally different while sharing a common evolutionary origin [40,43,44].

## Results

### In situ *B*. *burgdorferi* flagellar motor reveals novel structure of the ATPase complex

**Fig 1:**
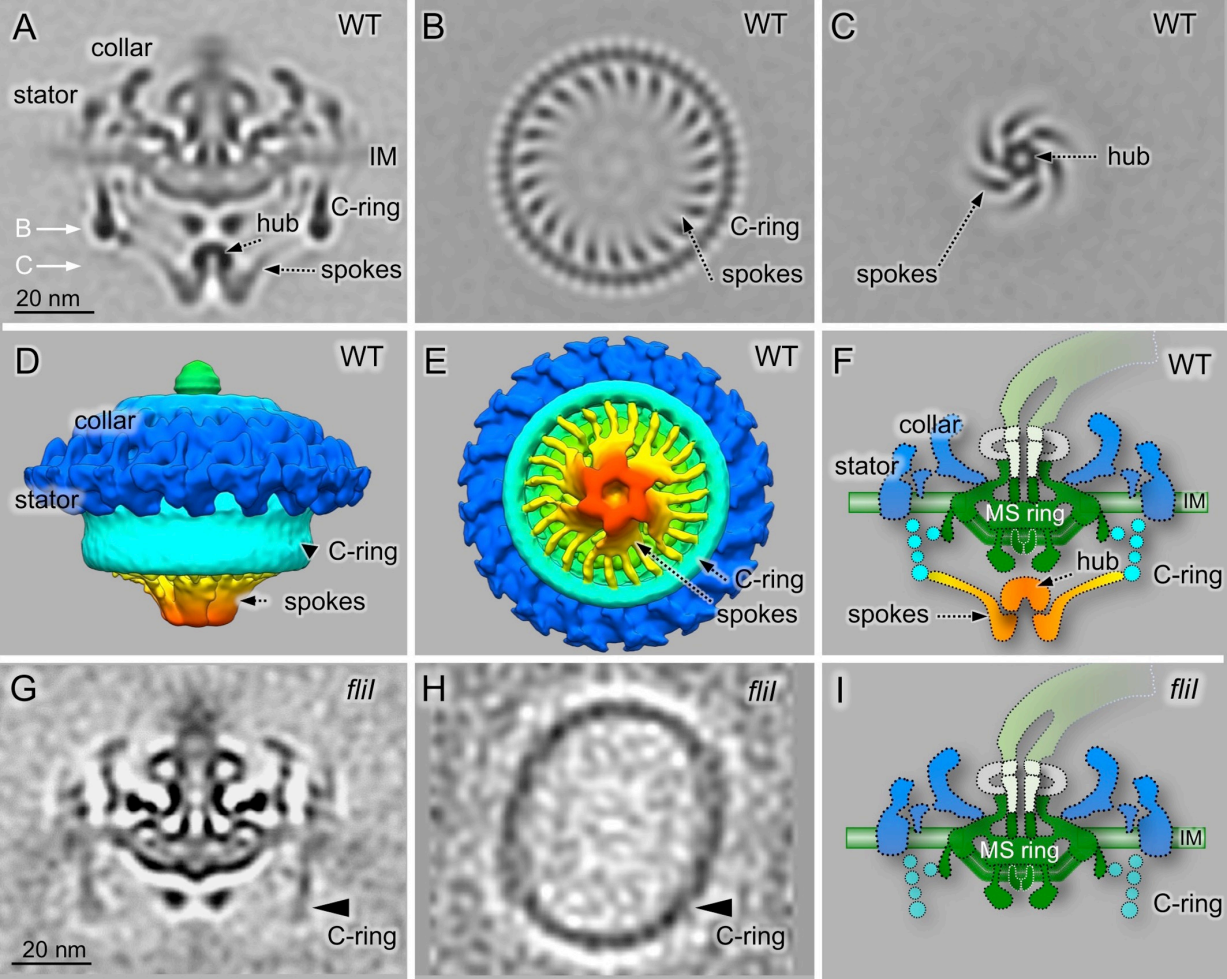
Cryo-ET reveals a novel ATPase complex structure in *B*. *burgdorferi*. (A) A central section of a flagellar motor structure from WT cells. The structure was generated after alignment of the ATPase complex region, classification of the spoke region (see S2 Fig), and image refinement on the C-ring and ATPase complex region. (B) A cross-section of the C-ring and spoke region. There are 23 spokes connecting the ATPase complex to the C-ring and 46 units at the bottom of the C-ring. (C) A cross-section of the ATPase region, showing the hexagonal “hub” densities. (D and F) Surface rendering of the WT flagellar motor from side and bottom, respectively. (F) A schematic model of the *B*. *burgdorferi* flagellar motor based on the averaged structure showed in (A). (G) A central section of the flagellar motor structure from a *fliI* mutant. The C-ring density from the *fliI* mutant is not well resolved compared to that from the WT (A). (H) A cross-section of the C-ring from approximately 50% of the *fliI* mutant shows an ellipse-like structure, which is very different from the C-ring in the WT flagellar motor in (B). (I) A schematic model of the flagellar motor structure in the *fliI* mutant. cryo-ET, cryo-electron tomography; WT, wild-type.

We utilized high-throughput cryo-ET and sophisticated subtomogram classification [45] to study the structure of *B*. *burgdorferi* flagellar motors. By analyzing 7,242 intact motor structures extracted from tomographic data collected on a direct detection device (DDD), we generated an asymmetric reconstruction that not only revealed the previously observed 16-fold symmetry of the collar and stator structures [10,11,39] but also disclosed a novel spoke-like structure underneath the C- and MS-rings (S1 Fig and S1 Movie). There are 23 spokes in most WT *B*. *burgdorferi* flagellar motors, albeit this number is varied from 21 to 24 in some rare instances (see S2 Fig). The spoke-like densities extend from a hexagonal “hub” to the bottom of the C-ring (S1 Fig and S1 Movie). We selected the class averages exhibiting 23 spokes, combined them, and did further image alignment with focus on the region of the “hub” and the C-ring (Fig 1A, 1B and 1C). The spokes extend from the central hub to the C-ring with 46-fold symmetry (Fig 1B).

FliI, FliH, and FliJ are known to form a large ATPase complex that was previously proposed to be centered on the FliI_6_ hexamer [25–27] and to correspond to the density underlying the FlhA ring based on analysis in *Campylobacter jejuni* [14] and in *B*. *burgdorferi* [42]. We speculate that the FliI_6_–FliJ complex forms the hexagonal hub and FliH is responsible for the spoke (Fig 1A, 1B and 1C). Indeed, our structures derived from *fliH* and *fliI* mutants [42] show that both the hub and the spokes are absent (Fig 1G), confirming that the distinct “hub and spoke” structure is dependent upon the presence of both FliI and FliH.

### The ATPase complex has profound impact on the C-ring structure

Our data showed that the ATPase complex is directly connected to the C-ring in the WT flagellar motor. The C-ring from the WT motors mostly maintained the round shape, with the aspect ratio ranging from 1.005 to 1.048 (S3A Fig). In contrast, the C-ring density in the *fliI* mutant is often more elliptical, with the aspect ratio ranging from 1.075 to 1.206 (S3B Fig). Among the *fliI* mutant motors, 49.9% have elliptical C-rings with aspect ratios ≥1.129, and 19.3% have more ellipse-shaped C-rings with aspect ratios ≥1.206. Therefore, we propose that the FliI–FliH complex plays an essential role in stabilizing the round-shaped C-ring structure. Without the support from the ATPase complex, the C-ring would lose the rigidity and become more flexible.

### Molecular architecture of the ATPase complex in *B*. *burgdorferi*

**Fig 2:**
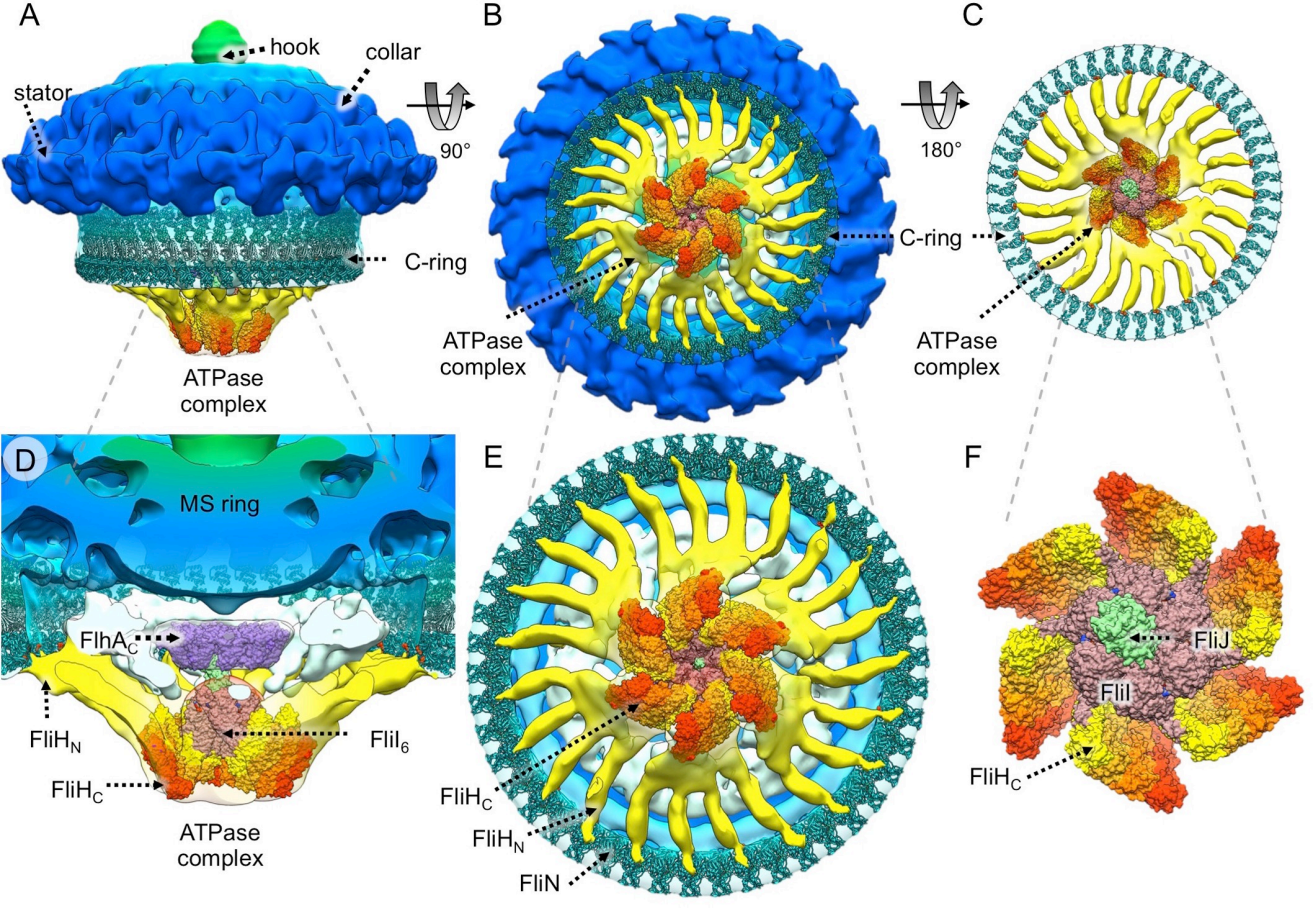
Proposed molecular architecture of the ATPase complex in the flagellar motor. Atomic structures of FliH, FliI, and FliJ were fitted into the cryoET-derived density map of the large hexametric complex attached to the C-ring protein FliN through the FliH spokes. As the C-ring shows 46-fold symmetry, 46 copies of the FliN tetramer as well as 46 copies of the FliG_MC_–FliM_M_ complex were placed into the C-ring. (A) A side view of the structure of the WT flagellar motor with the assembled C-ring (FliG, FliM, and FliN) and the ATPase complex (FliH, FliI, and FliJ). (B) A bottom-up view of the C-ring and the ATPase complex. (C) A top-down view of the assembled C-ring and the ATPase complex. (D) A sliced, enlarged view of the ATPase complex and its interactions with FlhA and FliN. (E) An enlarged, bottom view of the assembled C-ring and the ATPase complex. The hydrophobic surface (formed by Val-128, Val-129, and Val-130) of FliN interacts with the FliH spoke (yellow). (F) A close-up, top-down view of the assembled ATPase complex in which six FliI monomers form the “hub” and at least 23 FliH dimers form the spokes. cryo-ET, cryo-electron tomography; WT, wild-type.

To better understand the interactions between the ATPase complex and the C-ring in the intact *B*. *burgdorferi* flagellar motor, we constructed a model of the ATPase complex and its surrounding C-ring complex based on the available homologous structures. The crystal structures of FliI and FliJ from *Salmonella* [30,46] fit well into the central hub (Fig 2D and S4 Fig). The N- and C-termini of FliJ insert into the middle of six FliI subunits, while the middle part of FliJ inserts into the middle of the nonameric FlhA_C_ ring (Fig 2D and S4 Fig).

A FliH dimer (FliH_2_) is known to form a stable complex with the FliI ATPase [46,47]. The C-terminal domain of FliH is involved in binding to FliI, while a small central region of FliH is essential for formation of the FliH_2_ [48]. The N-terminal domain is important for FliH–FliN interactions [34, 35]. In our map, there are three or four spokes extending from each FliI monomer to the C-ring, although only one FliH_2_ binding site on each FliI was reported in a crystal structure of the FliI–FliH complex [46]. Therefore, we speculate that the first FliH_2_ dimer directly binds to one FliI monomer, while others bind to adjacent FliH_2_ dimers in a parallel fashion (S5 Fig); since FliI forms a monomeric hexamer, six bundles of FliH_2_ are attached to the central FliI hexamer in our model (Fig 2E and 2F, S5 Fig). The hydrophobic patch (L85, T110, V128, V130, F135) at the C-terminus of FliN has been reported to interact with FliH [34]. Our data indicate that the FliH_2_ spoke is indeed attached to FliN at bottom of the C-ring (Fig 2E), in which the atomic models of FliG, FliM, and FliN [49] were fitted into the C-ring density. With an additional rotation of approximately 10° from the initial model [49], the hydrophobic residues of FliN (labeled red in Fig 2D and S2 Movie) are located at the interface between FliN and the FliH spoke.

### The C-ring rotates with the ATPase complex

**Fig 3:**
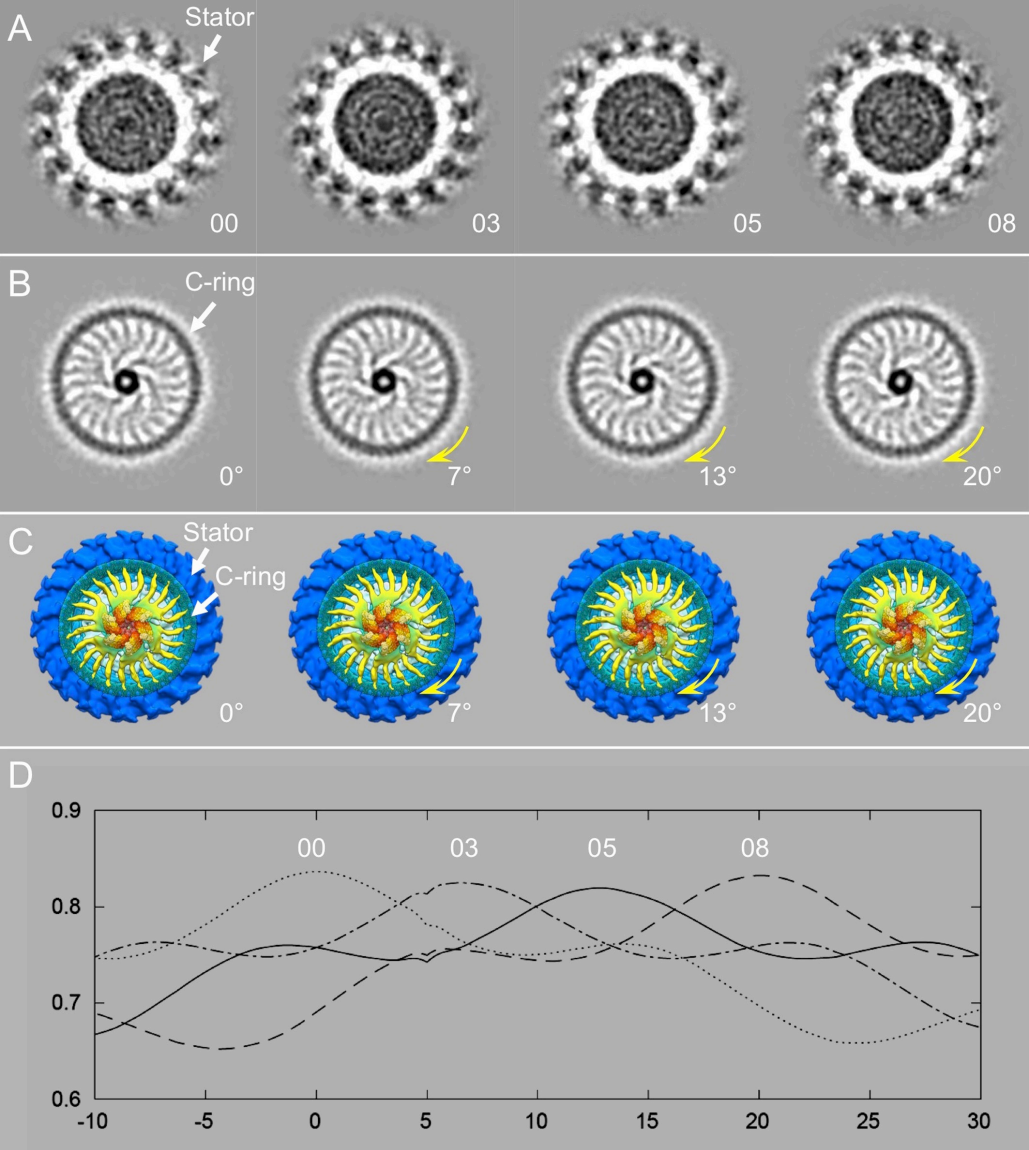
The ATPase complex adopts a different spin rotation in respect to the collar and the stators. (A) Sections of four class averages at the level of the 16 circumferential stator densities. Note that the stator densities exhibit very similar patterns on spin rotation. (B) Sections of the same class averages shown in panel A but taken at the level of the FliI/FliH assembly and the C-ring. The sections show the ATPase complex in slightly different orientations. There are different rotations in classes 03, 05, and 08 relative to class 00. (C) Cytoplasmic views of the ATPase complexes from the four class averages, corresponding to the cross-sections in panel B, respectively. (D) CCC plotting of the class averages. Note that the peak of the CCC for class 00 happens at 0° (without any in-plane rotation). The CCC peak for class 03 is located at approximately 7°; the CCC peak for class 05 is at approximately 13°, whereas the CCC peak for class 08 is at 20°. CCC, cross correlation coefficient.

The C-ring is thought to rotate together with the MS-ring and the flagellar filament, although the rotation of the C-ring has never been directly visualized. Here, because FliH spokes connected to the C-ring are visible, they can be utilized to track the rotation of the C-ring. Indeed, classification of the spoke region resulted in multiple structures, in which the ATPase complex apparently adopts different spin rotation with respect to the collar and the stator (see Fig 3 and S3 Movie). In the four classes shown in Fig 3, the cross-section view on collar and stator shows that those from four classes are in a similar orientation (Fig 3A); however, the cross-section view on the ATPase complex shows the spokes in classes 03, 05, and 08 rotate about 7°, 13°, and 20° from class 00 (Fig 3B), respectively. As the small angular change is difficult to discern, the class averages were analyzed by rotational cross correlation, and the coefficient was plotted (Fig 3D), showing the angular differences (7°, 13°, 20°) between class 00 and class 03, 05, and 08. As the spokes are attached to the C-ring, we propose that the C-ring and the ATPase can rotate together as a rigid body (see also in S3 Movie). Although the class average structures in Fig 3 were arranged as counter-clockwise (CCW) from left to right, the rotation can be either CCW or clockwise (CW).

## Discussion

**Fig 4:**
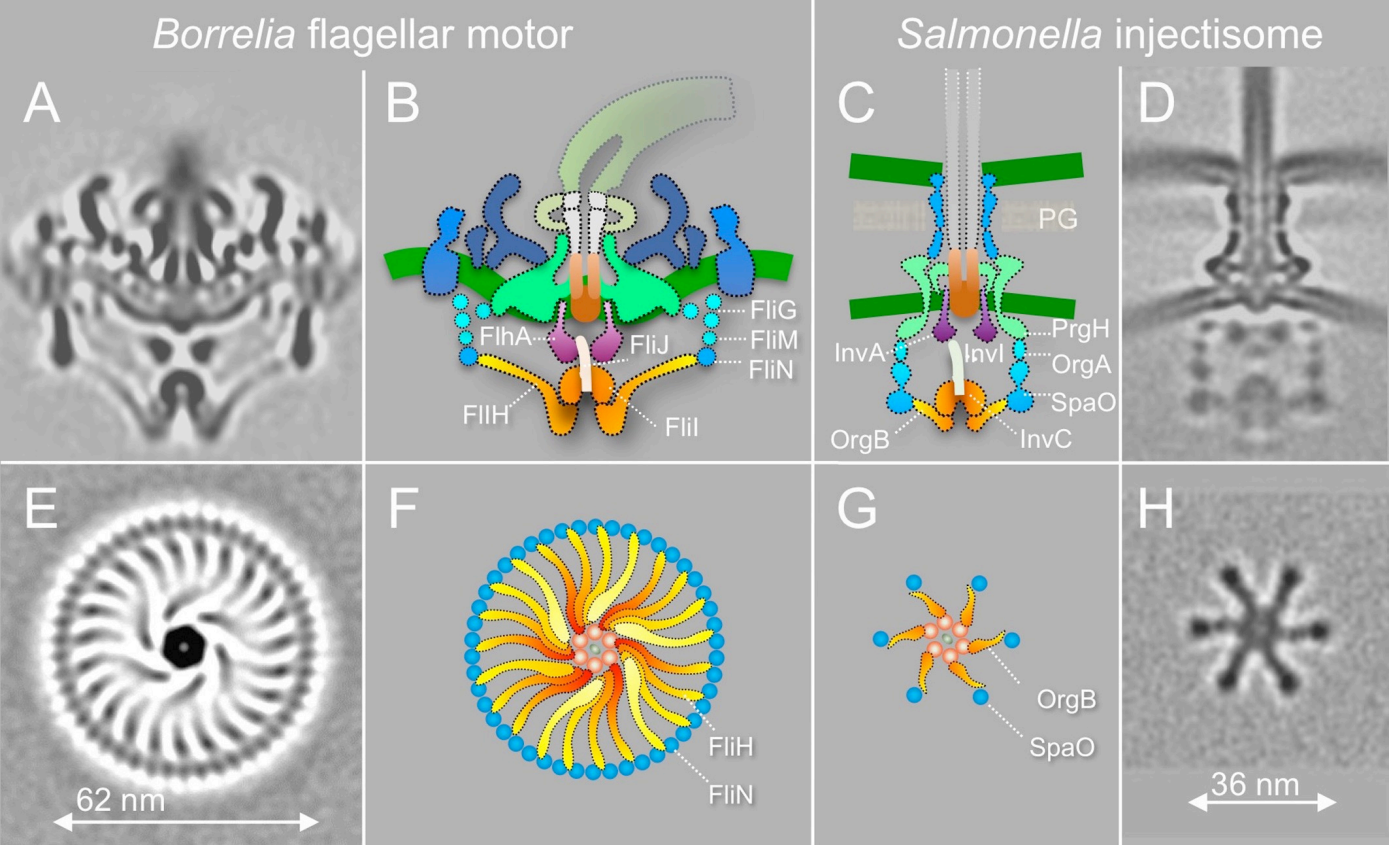
Comparison of the fT3SS from *B*. *burgdorferi* and the vT3SS from *Salmonella*. (A) A central section from the *B*. *burgdorferi* motor. (B) The fT3SS in the spirochete motor consists of the ATPase complex (orange) and the export apparatus (purple) underneath the MS-ring. (C, D) The vT3SS from *Salmonella* injectisome is modeled in a similar color scheme. The difference between the two T3SSs is striking in a comparison of the cross-sections of their ATPase complexes. Note that the C-ring from the *B*. *burgdorferi* motor is a continuous ring with approximately 46 copies of FliN tetramer. There are 23 visible FliH spokes (E, F). There are six pods in *Salmonella* injectisome. Only six spokes of the FliH homolog OrgB connect the ATPase complex to the SpaO molecules that compose the pod of the injectisome. fT3SS, flagella-specific Type III secretion system; vT3SS, virulence T3SS.

T3SSs in bacterial flagella and injectisomes are highly conserved and evolutionally related. The flagella are elaborate self-assembling machines that serve as the main organelles for bacterial motility. The injectisomes are specialized nanomachines deployed by many important human pathogens such as *Salmonella* spp., *Shigella* spp., and *Pseudomonas* to deliver virulence effectors into eukaryotic cells. Our previous studies revealed key intermediates of fT3SS-mediated assembly in *B*. *burgdorferi* [39] and overall architectures of the vT3SS machines in *Shigella* and *Salmonella* [43,44]. Here, we focus on in situ structure of the fT3SS machine in periplasmic flagella and compare it with external flagella and vT3SS machines. The overall organization of the fT3SS machine in the *B*. *burgdorferi* periplasmic flagella shares many similar features observed with the fT3SS machine in the *E*. *coli* external flagella [50] and the vT3SS machines in *Shigella* and *Salmonella* [40,43,44] (Fig 4). However, the ATPase complex of the *B*. *burgdorferi* periplasmic flagella is noticeably different from those observed in the *Salmonella* injectisome (Fig 4) and the *E*. *coli*/*Salmonella* external flagellum [40,50]. We observed 23 spokes and one hub in the ATPase complex of the *B*. *burgdorferi* periplasmic flagella. In contrast, no spoke has been observed in *E*. *coli*/*Salmonella* external flagellum. Only six spokes and one hub were observed in the *Salmonella* injectisome. The spokes are considerably longer in the *B*. *burgdorferi* flagellar basal body than in the *Salmonella* injectisome (6 nm versus 3 nm), consistent with the observation that the C-ring is much larger than the six “pods” (62 nm versus 36nm in diameter) (see Fig 4). Previous studies provided evidence that OrgB (a FliH homolog) forms the spoke-like structure and interacts with the ATPase complex and SpaO (a FliN homolog) of the *Salmonella* injectisome [43]. In the *B*. *burgdorferi* flagellar motor, the spoke between the ATPase and C-ring is likely formed by multiple FliH_2_ molecules. FliH of *B*. *burgdorferi* is significantly larger (305 amino acid residues) than its homolog in *Salmonella* (170 residues) (S6 Fig). Thus, the ATPase complex in the *B*. *burgdorferi* periplasmic flagella not only facilitates substrate recruitment and secretion but also supports the integrity of the C-ring, which undergoes rotation and switches between CW and CCW.

We observed many different orientations of the ATPase complex relative to the periplasmic structures of the motor, suggesting that the C-ring and the ATPase complex rotate together with the MS-ring. The rotation of the C-ring is driven by 16 stators that surround the C-ring and a spirochete-specific periplasmic collar [10]. In contrast, although OrgB and SpaO likely undergo high turnover with a cytoplasmic pool, the pods found in *Salmonella* injectisomes do not appear to rotate. The differences between the flagellar motor and injectisome underline the distinct mechanisms involved in their assembly and function.

Recent studies using fluorescence recovery after photobleaching showed the copy number of the C-ring protein FliN varies with the direction of flagellar rotation [51]. It was estimated in *E*. *coli* that there are 114 ± 17 FliN molecules in motors that rotated only CW and 144 ± 26 FliN in CCW motors [51]. Our multivariate statistical analysis results suggested that the FliH spoke numbers vary from 21 to 24 in the *B*. *burgdorferi* motor, and the spokes are distributed evenly along the C-ring (S1 Fig). If there are 46 FliN tetramers when there are 23 FliH spokes, there could be 42 FliN tetramers and 48 FliN tetramers when the spoke number is 21 and 24, respectively. Considering the C-ring in *B*. *burgdorferi* is relatively larger than the *E*. *coli* motor (57 nm versus 44 nm in diameter) [50], our estimation on FliN tetramers copies fall into a reasonable range compared with the observation from the *E*. *coli* motor. Yet further study on the ATPase complexes from the motors locked in CW or CCW rotation will be needed for a better understanding of the mechanisms underlying the C-ring proteins turnover and rotation.

In conclusion, our study reveals unprecedented details about the intact flagellar motor and its T3SS machine in the Lyme disease spirochete *B*. *burgdorferi*. We present the direct structural evidence that the flagellar ATPase complex is attached to the C-ring through multiple spokes likely comprised of FliH. The novel architecture of the ATPase complex not only strengthens the C-ring but also enables an optimal translocation of substrates through the ATPase complex and the export apparatus. Remarkably, the ATPase complex together with the C-ring can adopt variable orientations, implying that the fT3SS machine undergoes rotation with the flagellar C-ring. Together, our studies not only provide a structural framework for a better understanding of the fT3SSs but also underscore the striking differences between flagella and their evolutionally related bacterial injectisomes.

## Materials and methods

### Bacterial strains and growth conditions

High-passage *B*. *burgdorferi* strain B31A (WT) were grown at 35°C in BSK-II liquid medium supplemented with 6% rabbit serum or on semisolid agar plates in the presence of 2.5% carbon dioxide, as previously described [39,52].

### Frozen hydrated EM sample preparation

The frozen hydrated specimens were prepared as previously described [11]. Briefly, *B*. *burgdorferi* cultures were centrifuged at 5,000 × g for 5 min, and pellets were suspended in 1.0 ml phosphate buffered saline (PBS). The cells were centrifuged again and suspended in approximately 50–80 μl PBS. The cell suspensions were mixed with 10 nm colloidal gold and were then deposited onto freshly glow-discharged, holey carbon grids for 1 min. Grids were blotted with filter paper and then rapidly frozen in liquid ethane using a homemade gravity-driven plunger apparatus.

### Cryo-electron tomography

Frozen hydrated specimens were imaged at −170°C using a Polara G2 electron microscope (FEI) equipped with a field emission gun and a Gatan K2 Summit DDD. SerialEM was used to collect tilt series from WT cells in the dose fractionation mode [53]. The microscope was operated at a magnification of 15,400×, resulting in an effective pixel size of 2.5 Å without binning and a cumulative dose of approximately 60 e^−^/Å^2^ distributed over 61 stacks. Each stack contains eight images. Tomoauto was utilized to facilitate the automation of cryo-ET data processing [44]. The main executables include the following: drift correction of dose-fractionated data using Motioncorr [54] and assembly of corrected sums into the tilt-series, alignment of tilt-series and CTF correction by IMOD [55], and reconstruction of tilt-series into tomograms by TOMO3D [56].

### 3D image processing and subtomogram averaging

In total, we extracted 7,242 motors from 780 tomograms on WT cells. The subtomogram analysis was implemented as previously described [11,39,57]. Briefly, the initial orientation of each motor was estimated by the center coordinates of the flagellar C-ring and the collar, thereby providing two of the three Euler angles. To accelerate image analysis, 4×4×4 binned subtomograms (64×64×64 voxels) were used for initial alignment. Then, the original subtomograms (256×256×256 voxels) were utilized for further image analysis. Multivariate statistical analysis and hierarchical ascendant classification were then applied to analyze the intact motor [45,58,59]. Relevant voxels of the aligned subvolumes were selected by specifying a binary mask of the motor. Class averages were computed in Fourier space, so the missing wedge problem of tomography was minimized. All class averages were further aligned with each other to minimize differences in motor orientation.

Because the symmetric feature of the collar and stators is predominant, initially, the averaged structure of motors showed the 16-fold symmetry of those regions, while the structure of the ATPase complex is not well resolved. Classification on the ATPase complex yielded several class averages with 6-fold symmetry. The class averages with obvious 6-fold symmetry were selected for further analysis. The 5,076 subtomograms in this data set were aligned on the ATPase complex region by spin alignment with step size of 22.5° (360°/16). As a result, in the global average, the “hub” showed evident features with 6-fold symmetry, while the periplasmic features maintained 16-fold symmetry.

The average structure of the spokes from WT cells was generated as follows: (1) the 5,076 selected subtomograms that give 16-fold symmetry in the stator and collar regions and 6-fold symmetry in the “hub” region of the average structure were classified based on the spoke region. This analysis generated four class averages, three of which showed spokes. One of those three class averages is presented in S1 Movie, illustrating the 3D distribution of the 16-fold symmetry at collar and stator regions, 23-fold symmetry of the spokes, and 6-fold symmetry of the “hub.” The structure in S1 Movie shows that three regions with different symmetry can be resolved in one averaged structure from real data. (2) Classification on the spoke region was carried out using eigenimages one to 18 to generate four class averages (see S2 Fig). The first 40 eigenimages of the data set indicate the presence of different symmetries of the spoke region (see Results).

To define the rotation angles, the previously aligned subtomograms were classified on the collar and stator regions. The new class averages that showed symmetry of the collar and stator regions were selected and aligned by the collar and stator regions with spin alignment only. The spin rotation angles were recorded and compared.

### 3D visualization and modeling

UCSF Chimera [60] was used for 3D visualization of flagellar motors. The crystal structure of MxiA_C_ (PDB: 4A5P) was fitted directly into the tomographic density map of the FlhA region. The FliI/FliJ model based on two crystal structures from *Salmonella* [30,46] was fitted into the hexagonal “hub.” The atomic structure of FliI–FliH_2C_ (PDB:5B0O) was initially fitted into the segmented density by rigid fitting. As there is extra density for FliH_2_ in the tomographic map and three to four spokes extend from the “hub,” three more FliH_2_ were placed adjacent to the first FliH_2_; they were fitted into the density map using MDFF [61] (see S2 Movie).

The crystallographic structure of *E*. *coli* FliN is organized in doughnut-shaped tetramers [62]. Combined with a recent crystal structure FliM_M_–FliG_MC_ complex from *Thermotoga maritima* (PDB:4FHR) [49], the FliN–FliM_M_–FliG_MC_ complex fits well into the bulge density at the bottom of the C-ring (S2 Movie). As V111, V112, and V113 (*E*. *coli*) are in the hydrophobic patch and interaction with FliH [63], we speculate that those three valine residues face toward the FliH_2_ spoke. Those three valines correspond to V128, V129, and V130 in *T*. *maritima* [63]. As a result, when we fit the FliN tetramer ring, we have V128, V129, and V130 (See S2 Movie shown in red) facing toward the FliH_2_ spoke. There are 46 copies of FliG–FliM–FliN, and they fit reasonably well into the *B*. *burgdorferi* C-ring density.

## Supporting information

S1 Fig Asymmetric reconstruction of the flagellar motor from *B*. *burgdorferi*. (A) A central section of the averaged structure. (B–F) Different cross-sections show variable symmetries from the top to the bottom of the flagellar motor, respectively. The location of each cross-section is shown in panel A.
(JPG)

S2 Fig Main eigenimages for the classification on the spoke region. The first 40 eigenimages of the data set show different symmetry of the spoke region. Eigenimages 01 and 02 exhibit 23-fold symmetry. Eigenimages 04 and 07 exhibit 22-fold symmetry. Eigenimages 08 and 09 exhibit 21-fold symmetry. Eigenimages 10 and 11 exhibit 24-fold symmetry.
(JPG)

S3 Fig Comparison of the flagellar C-ring from WT and *fliI* mutant. *B*. *burgdorferi* flagellar motors from WT and *fliI* mutant were aligned and classified on the C-ring. (A) Top: cross-sections of four averages from WT. Bottom: the red circle superimposed on the C-ring measures the aspect ratio of each class average. The ratio and the percentage of motors in each class are shown below the class averages. (B) Top: cross-sections of four class averages from the *fliI* mutant. Bottom: the red circle superimposed on the C-ring measures the aspect ratio of each class average. The ratio and the percentage of motors in each class are shown below the class averages. WT, wild-type.
(JPG)

S4 Fig The modeling of the ATPase complex. (A) The segmentation of the bell-shaped density shows multiple spokes (yellow) and six symmetric densities (orange) around one extra density in the middle (light green). (B) A model of the FliI–FliJ complex based on two crystal structures of FliI (PDB:5B0O) and FliJ (PDB:3AJW) from *Salmonella* fitted well into the segmented map, although the corresponding density of FliJ covers only its small fraction. (C) A side view of the segmented map and (D) the model after the fitting. PDB, Protein Data Bank.
(JPG)

S5 Fig A model of the FliI–FliJ–FliH complex in periplasmic flagella. (A) The crystal structure of FliI–FliH complex (PDB:5B0O). (B) Another FliH_C2_ (FliH_C2_-2) could bind to the first FliH_C2_ (FliH_C2_-1). (C) Charge–charge interaction between the two FlH_C2_. One side of FliH_C_-A is positively charged with amino acid R179. The complimentary surface of FliHc-C is negatively charge with E161, D214, and D216. Additionally, one side of FliH_C_-D is positively charge with R104. The complementary surface of FliH_C_-B is negatively charged with D175, D198, E181, and E182. (D) The interaction surface between FliH_C2_-1 and FliH_C2_-2. (E) Hydrophobic surface (yellow) between the two FlH_C2_. FliHc-C and FiH_C_-D binds to the C1 α-helix of FliH_C_-B (L215 to C227) through hydrophobic interaction. The hydrophobic groove was formed by α1b, α1a’, and α1b’ (see C). The hydrophobic residues include I123, I127, and A131 of FliH_C_-C and A118, L119, V122, V123, V124, L127, M130, A134, I154, L157, L158, L163, F164, L229, and A230 from FliH_C_-D. (F) A model of FliI–FliH_C_ complex including one FliI and four FliH_C2_. (G) A model of the ATPase complex. PDB, Protein Data Bank.
(JPG)

S6 Fig Modeling of FliI–FliH complex. (A) The distance between P100 on FliH to V130 on FliN is approximately 20 nm. Using sequence alignment, we found P100 from FliH of *Salmonella* (CAD05719.1) is aligned with K170 from FliH of *B*. *burgdorferi* (AAA8612.1). There are 170aa of the *B*. *burgdorferi* FliH that could build the gap between K170 on FliH and V130 on FliN.
(JPG)

S1 Movie Asymmetric reconstruction of the *B*. *burgdorferi* flagellar motor reveals a 16-fold symmetric feature at collar and stator regions, 23 spokes, and a hexagonal hub. The left is the side view with yellow line slicing through; the right is the cross-section view corresponding to the yellow line. The movie shows distinct symmetries within the *B*. *burgdorferi* flagellar motor.
(MP4)

S2 Movie Surface rendering and modeling of the *B*. *burgdorferi* flagellar motor. The *B*. *burgdorferi* C-ring model was built based on a homology model from *Thermotoga maritime* (Vartanian and colleagues, 2012), with the hydrophobic patch of FliN tetramer facing the spokes. The *B*. *burgdorferi* ATPase complex was built based on two homologous structures from *Salmonella* (PDB:5B0O and PDB:3AJW). The FlhA cytoplasmic complex was built based on the structure from the homolog MxiA (PDB:4A5P). PDB, Protein Data Bank.
(MOV)

S3 Movie Multiple structures of the ATPase complex are assembled together to show the rotation of the ATPase complex and the C-ring relatively to the stator, which is known to be anchored to the cell wall. (MPEG)

## Abbreviations

CCW: counter-clockwise
cryo-ET: cryo-electron tomography
CW: clockwise
DDD: direct detection device
fT3SS: flagella-specific type III secretion system
PBS: phosphate buffered saline
PDB: Protein Data Bank
vT3SS: virulence T3SS
WT: wild-type
