# Supplementary figures and images for: Cryo-electron tomography of periplasmic flagella in Borrelia burgdorferi reveals a distinct cytoplasmic ATPase complex
Source: PLoS Biol. 2018 Nov 9;16(11):e3000050. doi: 10.1371/journal.pbio.3000050 (PMC6248999; doi:10.1371/journal.pbio.3000050)

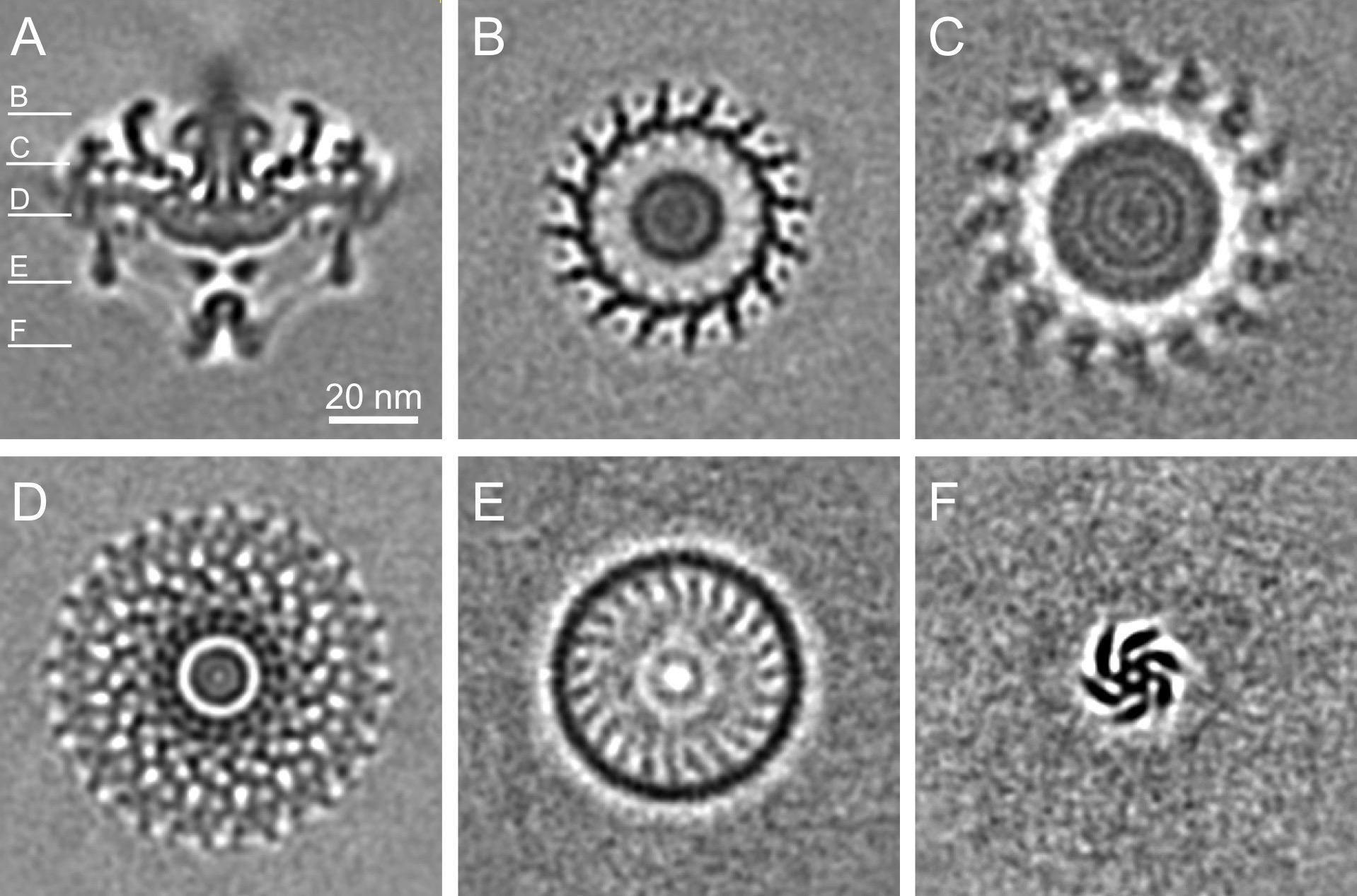

Supplement: S1 Fig — (A) A central section of the averaged structure. (B–F) Different cross-sections show variable symmetries from the top to the bottom of the flagellar motor, respectively. The location of each cross-section is shown in panel A. (JPG) [file pbio.3000050.s001.jpg]

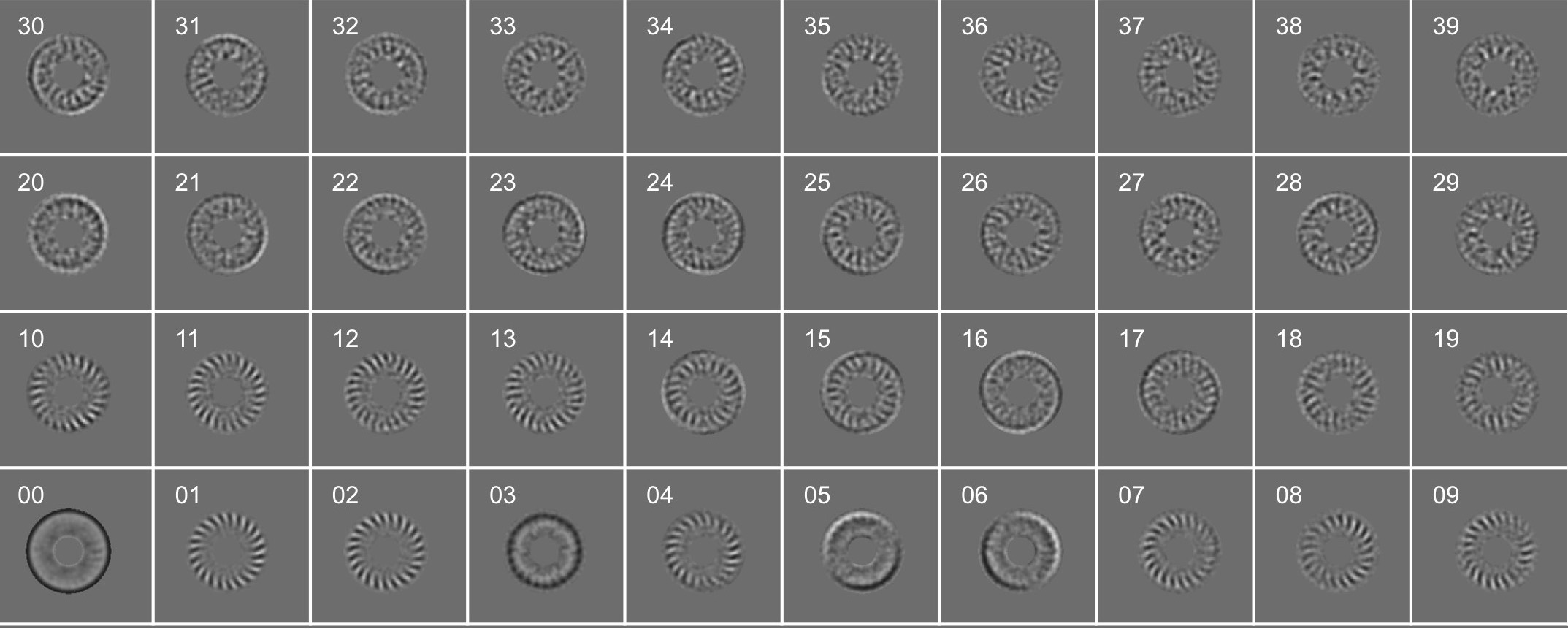

Supplement: S2 Fig — The first 40 eigenimages of the data set show different symmetry of the spoke region. Eigenimages 01 and 02 exhibit 23-fold symmetry. Eigenimages 04 and 07 exhibit 22-fold symmetry. Eigenimages 08 and 09 exhibit 21-fold symmetry. Eigenimages 10 and 11 exhibit 24-fold symmetry. (JPG) [file pbio.3000050.s002.jpg]

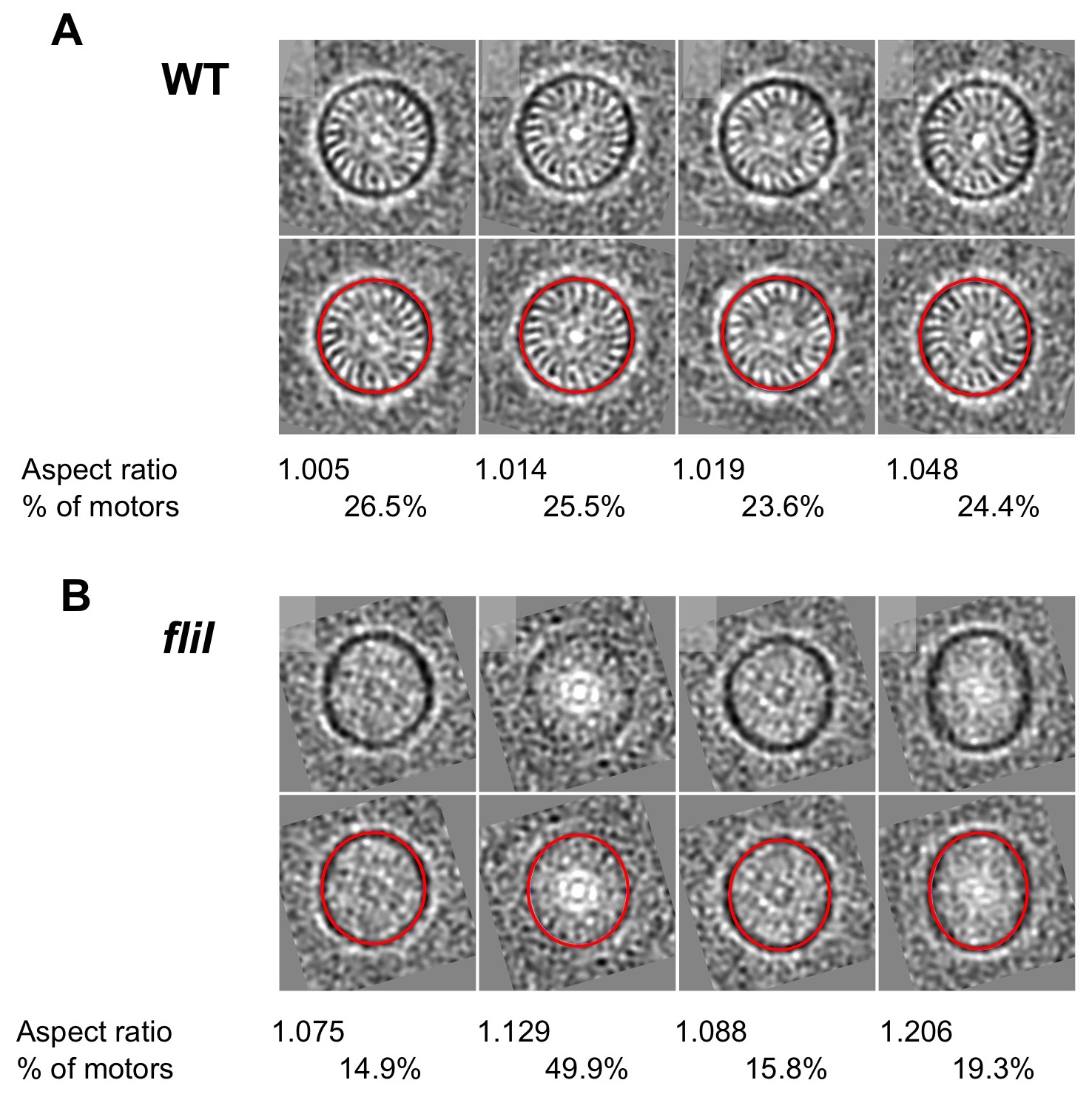

Supplement: S3 Fig — B. burgdorferi flagellar motors from WT and fliI mutant were aligned and classified on the C-ring. (A) Top: cross-sections of four averages from WT. Bottom: the red circle superimposed on the C-ring measures the aspect ratio of each class average. The ratio and the percentage of motors in each class are shown below the class averages. (B) Top: cross-sections of four class averages from the fliI mutant. Bottom: the red circle superimposed on the C-ring measures the aspect ratio of each class average. The ratio and the percentage of motors in each class are shown below the class averages. WT, wild-type. (JPG) [file pbio.3000050.s003.jpg]

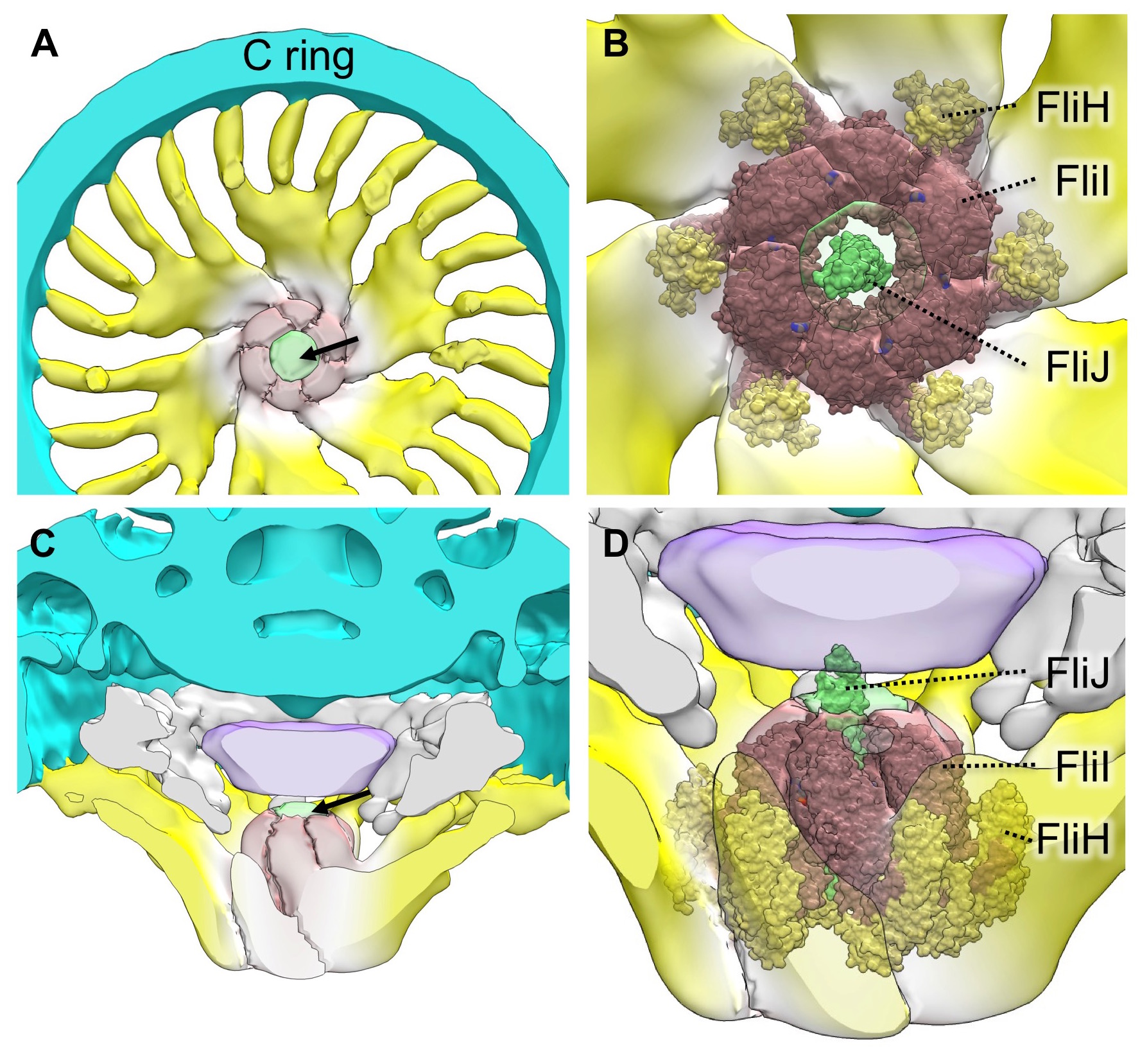

Supplement: S4 Fig — (A) The segmentation of the bell-shaped density shows multiple spokes (yellow) and six symmetric densities (orange) around one extra density in the middle (light green). (B) A model of the FliI–FliJ complex based on two crystal structures of FliI (PDB:5B0O) and FliJ (PDB:3AJW) from Salmonella fitted well into the segmented map, although the corresponding density of FliJ covers only its small fraction. (C) A side view of the segmented map and (D) the model after the fitting. PDB, Protein Data Bank. (JPG) [file pbio.3000050.s004.jpg]

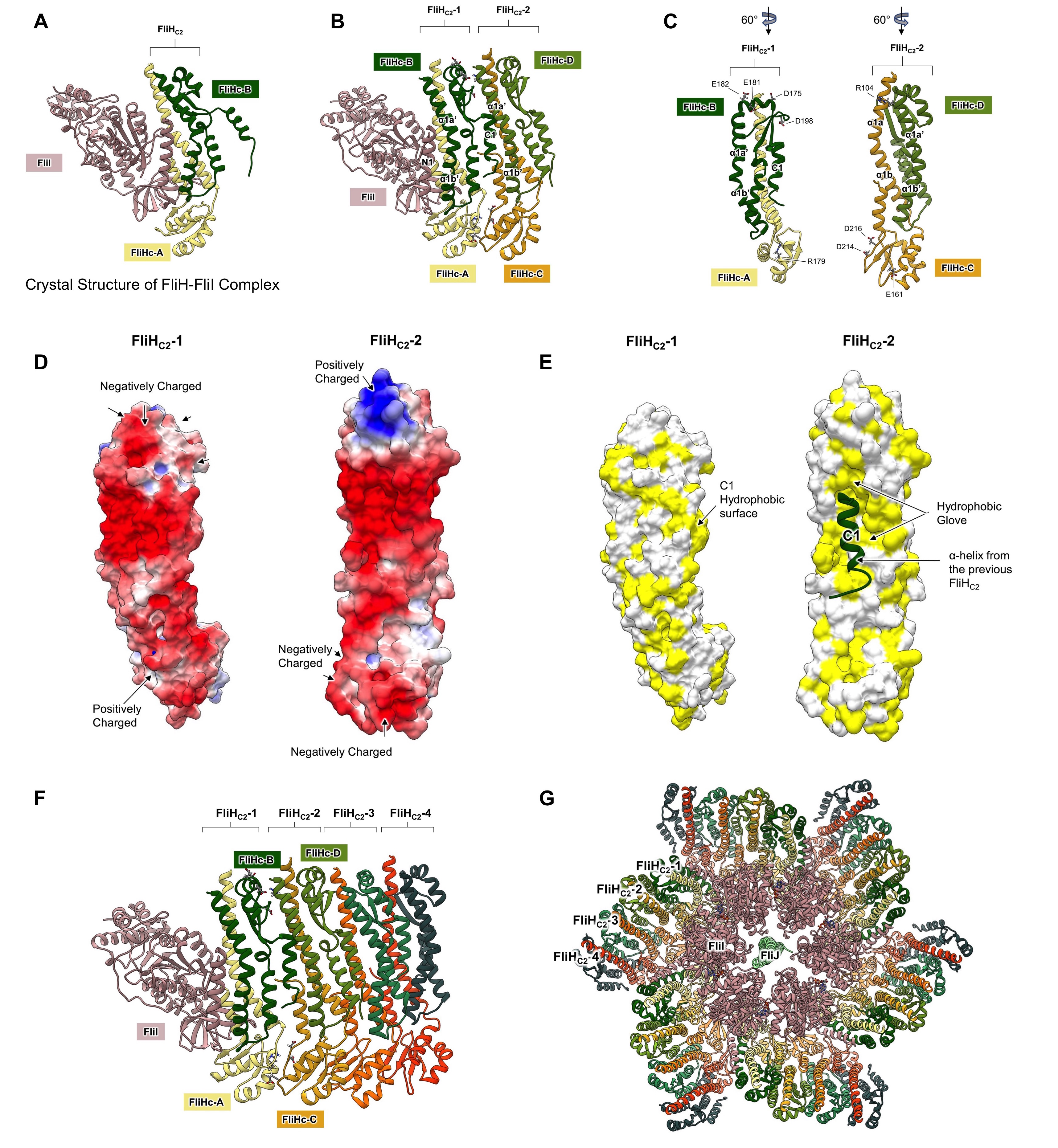

Supplement: S5 Fig — (A) The crystal structure of FliI–FliH complex (PDB:5B0O). (B) Another FliHC2 (FliHC2-2) could bind to the first FliHC2 (FliHC2-1). (C) Charge–charge interaction between the two FlHC2. One side of FliHC-A is positively charged with amino acid R179. The complimentary surface of FliHc-C is negatively charge with E161, D214, and D216. Additionally, one side of FliHC-D is positively charge with R104. The complementary surface of FliHC-B is negatively charged with D175, D198, E181, and E182. (D) The interaction surface between FliHC2-1 and FliHC2-2. (E) Hydrophobic surface (yellow) between the two FlHC2. FliHc-C and FiHC-D binds to the C1 α-helix of FliHC-B (L215 to C227) through hydrophobic interaction. The hydrophobic groove was formed by α1b, α1a’, and α1b’ (see C). The hydrophobic residues include I123, I127, and A131 of FliHC-C and A118, L119, V122, V123, V124, L127, M130, A134, I154, L157, L158, L163, F164, L229, and A230 from FliHC-D. (F) A model of FliI–FliHC complex including one FliI and four FliHC2. (G) A model of the ATPase complex. PDB, Protein Data Bank. (JPG) [file pbio.3000050.s005.jpg]

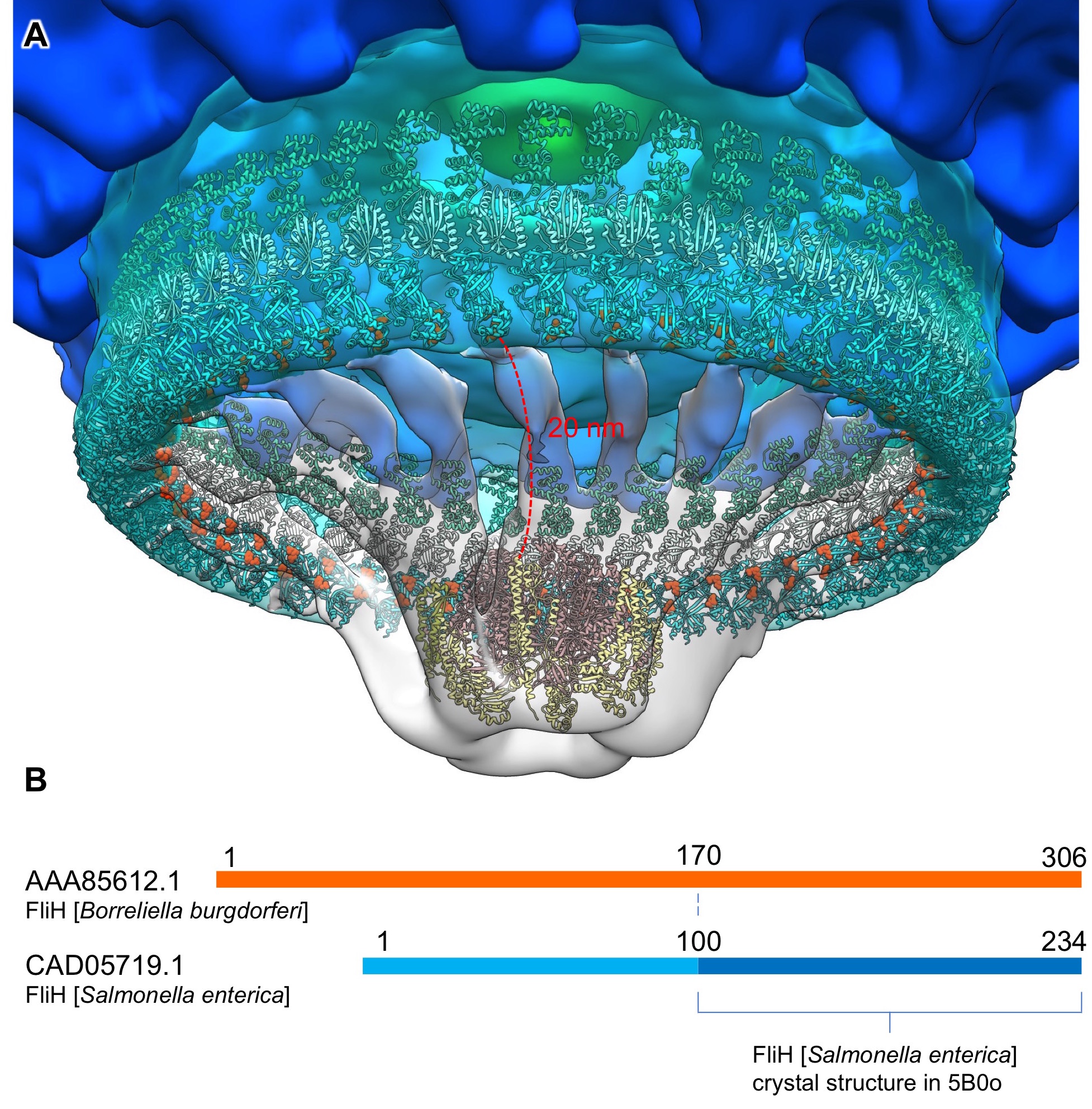

Supplement: S6 Fig — (A) The distance between P100 on FliH to V130 on FliN is approximately 20 nm. Using sequence alignment, we found P100 from FliH of Salmonella (CAD05719.1) is aligned with K170 from FliH of B. burgdorferi (AAA8612.1). There are 170aa of the B. burgdorferi FliH that could build the gap between K170 on FliH and V130 on FliN. (JPG) [file pbio.3000050.s006.jpg]
